# Transcriptomic Analysis Followed by the Isolation of Extracellular Bacteriolytic Proteases from *Lysobacter capsici* VKM B-2533^T^

**DOI:** 10.3390/ijms241411652

**Published:** 2023-07-19

**Authors:** Alexey Afoshin, Irina Kudryakova, Sergey Tarlachkov, Elena Leontyevskaya, Dmitry Zelenov, Pavel Rudenko, Natalya Leontyevskaya (Vasilyeva)

**Affiliations:** 1 Laboratory of Microbial Cell Surface Biochemistry, G.K. Skryabin Institute of Biochemistry and Physiology of Microorganisms, FRC PSCBR, Russian Academy of Sciences, 5 Prosp. Nauki, Pushchino 142290, Russia; alex080686@mail.ru (A.A.); kudryakovairina@yandex.ru (I.K.); sergey@tarlachkov.ru (S.T.); ealeont@gmail.com (E.L.); zelenovdima1@mail.ru (D.Z.); pavelrudenko76@yandex.ru (P.R.); 2 Pushchino Branch of the Federal State Budgetary Educational Institution of Higher Education «Russian Biotechnological University (BIOTECH University)», 3 Institutskaya Str., Pushchino 142290, Russia

**Keywords:** extracellular bacteriolytic enzymes, RNA-seq, *Lysobacter capsici*, homologous expression system, antimicrobial potency

## Abstract

The aim of the study was to search for, isolate and characterize new bacteriolytic enzymes that show promising potential for their use in medicine, agriculture and veterinary. Using a transcriptomic analysis, we annotated in *Lysobacter capsici* VKM B-2533^T^ the genes of known bacteriolytic and antifungal enzymes, as well as of antibiotics, whose expression levels increased when cultivated on media conducive to the production of antimicrobial agents. The genes of the secreted putative bacteriolytic proteases were also annotated. Two new bacteriolytic proteases, Serp and Serp3, were isolated and characterized. The maximum bacteriolytic activities of Serp and Serp3 were exhibited at low ionic strength of 10 mM Tris-HCl, and high temperatures of, respectively, 80 °C and 70 °C. The pH optimum for Serp was 8.0; for Serp3, it was slightly acidic, at 6.0. Both enzymes hydrolyzed autoclaved cells of *Micrococcus luteus* Ac-2230^T^, *Proteus vulgaris* H-19, *Pseudomonas aeruginosa* and *Staphylococcus aureus* 209P. Serp also digested cells of *Bacillus cereus* 217. Both enzymes hydrolyzed casein and azofibrin. The newly discovered enzymes are promising for developing proteolytic antimicrobial drugs on their basis.

## 1. Introduction

Of increasing importance in recent years have been the search for, isolation and characterization of new antimicrobial agents. Bacteriolytic enzymes are among such agents.

In bacterial peptidoglycan, bacteriolytic enzymes hydrolyze various bonds. Glucosaminidases cleave the bond between N-acetylglucosamine and N-acetylmuramic acid of peptidoglycan. Muramidases hydrolyze the bond between N-acetylmuramic acid and N-acetylglucosamine of peptidoglycan. Amidases cleave the bond between N-acetylmuramic acid and the first amino acid of the peptide subunit. Bacteriolytic proteases hydrolyze peptide bonds of peptidoglycan. 

The very first and best known and, presently, the most commercially available bacteriolytic enzyme, lysozyme, is a muramidase. Other bacteriolytic enzymes are also known, surpassing lysozyme in their activity and antimicrobial spectrum. Bacteriolytic proteases are among those enzymes. The best known are α- and β-lytic proteases described forty years after the discovery of the lysozyme [1]. They became the second and third discovered bacteriolytic enzymes. These enzymes were first isolated from the culture fluid of the Gram-negative bacterium *Lysobacter enzymogenes*. Currently, *Lysobacter* bacteria are among the best producers of antimicrobial agents, which, in addition to bacteriolytic enzymes, also include antibiotics and antimicrobial peptides [2,3,4]. Bacteriolytic enzymes of Gram-negative bacteria are not so widespread due to the complexity of their isolation, their insufficient coverage in studies and lack of biotechnologically valuable expression systems.

We have studied the antimicrobial potential of *Lysobacter capsici* strains for many years now. An antimicrobial drug, lysoamidase, based on the culture fluid of strain *Lysobacter* sp. XL1 (now *L. capsici* XL1), has been developed [5]. This drug contains a complex of bacteriolytic enzymes. Our recent research has investigated the lytic properties of strain *L. capsici* VKM B-2533^T^. This strain possesses potent antifungal and bacteriolytic activities. We have isolated α- and β-lytic proteases, lysine-specific proteases, as well as identified new bacteriolytic proteases of MW 26 and 29 kDa, including N-acetylglucosaminidase [6]. For the β-lytic protease, the spatial structure has been established and its characterization has been expanded; an effective expression system has been developed [7,8]. The development of this system will enable us to isolate and study bacteriolytic *Lysobacter* enzymes with high efficiency. During the purification of bacteriolytic enzymes from the culture fluid of *Lysobacter capsici* VKM B-2533^T^, we identified bacteriolytically active fractions containing protein mixtures. This was indicative of the presence of other bacteriolytic enzymes besides those already identified. To search for new bacteriolytic enzymes, we used a transcriptomic approach. 

## 2. Results

### 2.1. Search for the Genes of Bacteriolytic Enzymes Using the Transcriptomic Approach

**Figure 1:**
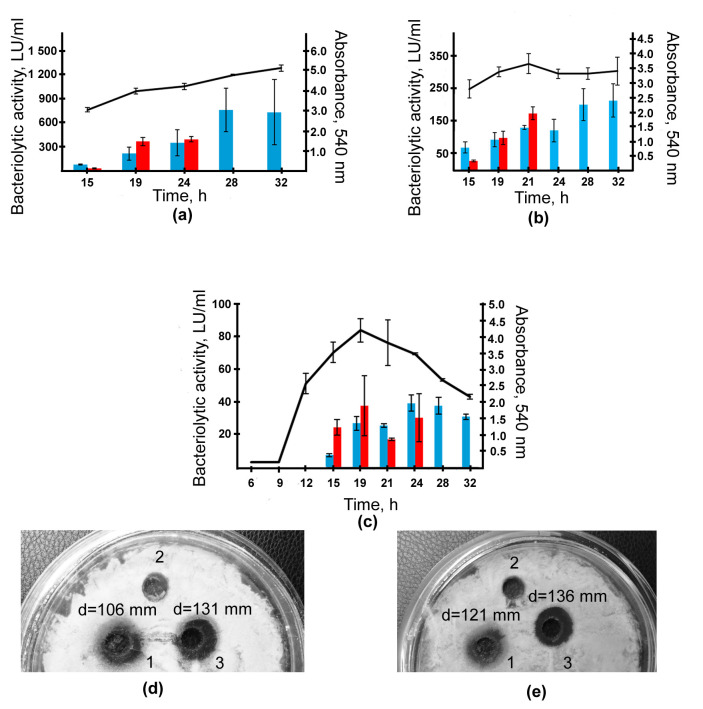
Dynamics of growth and bacteriolytic activity of *Lysobacter capsici* VKM B-2533^T^ cultivated on media RM (**a**), SYM (**b**) and 5/5 (**c**) for 32 h. Blue bars indicate the total bacteriolytic activity of the culture fluid with respect to autoclaved cells of *Staphylococcus aureus* 209P; red bars indicate the same with respect to living *S. aureus* 209P cells. The antifungal activity of the culture fluid after cultivation on media RM (1), 5/5 (2) and SYM (3) for 19 h with respect to *Fusarium solani* (**d**) and *Sclerotinia sclerotiorum* (**e**). Medium 5/5 was used as a negative control. d, Lysis zone diameter measured from the center of the well to the edge of the lysis zone.

Earlier, we have shown that the production of antimicrobial agents in *L. capsici* VKM B-2533^T^ depends on the culture medium [6]. In this strain, the maximum bacteriolytic activity was observed when grown on RM medium; the maximum antifungal activity was observed when grown on SYM medium. On medium 5/5, the lytic activity was minimal. For transcriptomic analysis, we decided to study the strain cells grown on these media. The results obtained with medium 5/5 were used as a negative control. To choose the optimal point of cell selection for transcriptomic analysis, we studied the dynamics of growth and bacteriolytic activity of the strain on the chosen media (Figure 1).

As shown in Figure 1a–c, the maximum bacteriolytic activity with respect to *S. aureus* 209P living cells was observed by 19–21 h of cultivation, to be 366 LU/mL, 99 LU/mL and 38 LU/mL on RM, SYM and 5/5 media, respectively. The maximum antifungal activity was observed by this time on SYM medium (Figure 1d,e). On all media, by 19 h of cultivation, the cells reached the end of the exponential growth phase. This phase of growth is characterized by the maximum biosynthetic activity. Thus, the point corresponding to 19 h of cultivation is optimal for obtaining the material for a transcriptomic study. As a result, the RNA was isolated from cells grown on the chosen media for 19 h (Supplementary File S1 Figure S1) and was sequenced (Section 4).

Analysis showed that all samples had sufficient sequencing depths, mostly greater than 10 million reads per sample. The lowest value is 9.9 million reads for a sample of SYM rep. 3; the highest was 14.1 million reads for a sample of RM rep. 1 following the trimming by quality and adapter removal. On average, 98.3% of the reads were successfully aligned to the reference genome of *L. capsici* VKM B-2533T, and 67.2% reads on average were uniquely assigned to the annotated genes. All sequencing and alignment statistics are shown in Supplementary File S1 Table S1.

Pearson *r*^2^ correlation values for all replicates were between 0.85 and 0.99, and a mean value for biological replicates was 0.98. A clustering tree of the samples also indicated the consistency of the obtained data (Supplementary File S1 Figure S2).

**Figure 2:**
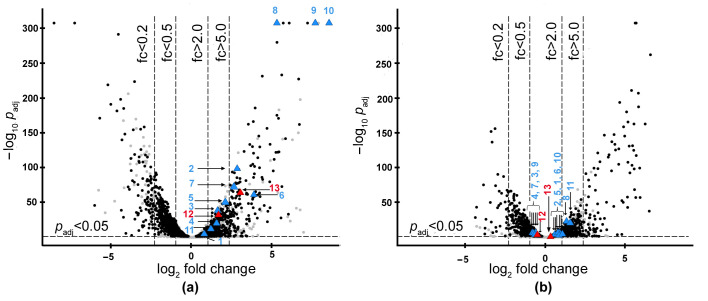
Volcano plots of gene expression for RM (**a**) and SYM (**b**) media. Genes that significantly changed their expression levels on both media are highlighted in black. 1, Locus tag IEQ11_03495 (UOF15745.1); 2, IEQ11_04180 (UOF15870.1); 3, IEQ11_06885 (UOF16369.1); 4, IEQ11_09745 (UOF16892.1); 5, IEQ11_12530 (UOF17397.1); 6, IEQ11_14225 (UOF12917.1); 7, IEQ11_15570 (UOF13168.1); 8, IEQ11_15580 (UOF13170.1); 9, IEQ11_17420 (UOF13513.1); 10, IEQ11_22400 (UOF14439.1); 11, IEQ11_23755 (UOF14691.1); 12, IEQ11_08595 (UOF16681.1); 13, IEQ11_14490 (UOF12968.1). Blue and red triangles, the genes of interest. Red triangles, the genes chosen to be studied.

Analysis of differentially expressed genes (DEGs) revealed 3912 genes whose expression levels changed on at least one medium compared to the control (*p*_adj_ < 0.05). Of these, 1961 genes changed expression on both media (Figure 2, Supplementary File S2).

**Figure 3:**
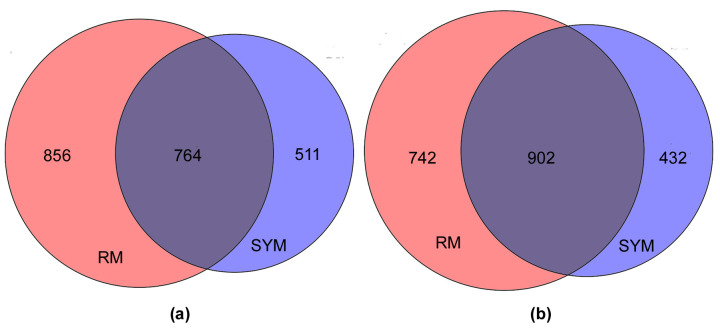
Area-proportional Venn diagrams showing the number of DEGs that overlapped between RM (red) and SYM (blue) media. The diagrams show the number of induced (**a**) and repressed (**b**) genes, respectively.

The number of induced genes is approximately equal to the number of repressed genes (*p*_adj_ < 0.05) (Figure 3).

First of all, we analyzed the gene expression of the known bacteriolytic enzymes identified earlier in strain VKM B-2533^T^ [6]. These are the enzymes Blp, Serp, L1, Serp6, Serp7, and N-acetylglucosaminidase. We also searched for the genes of bacteriolytic enzymes L4 and L5 known for *L. capsici* XL1 but not annotated earlier in strain VKM B-2533^T^. The results of the analysis are presented in Table 1. The required genes are also marked with triangles in Figure 2.

As shown in Table 1, all required genes of the bacteriolytic enzymes were identified. A noticeable increase in the expression of bacteriolytic enzyme genes is observed in the cultivation of the strain on RM medium, which is consistent with the LU/mL data. The greatest increases in expression were 7-, 39- and 6-fold, respectively, on this medium, which were shown for the enzymes Blp, L1 and L5. On SYM medium, the expression of bacteriolytic enzyme genes increased only slightly, and a decrease in expression was shown for the genes of the L4, L5 and Serp enzymes. These results are also consistent with the LU/mL data.

Analysis also revealed that among the entire pool of genes annotated as proteolytic enzymes, the greatest increase in expression was observed for metalloprotease and serine protease genes, 46% and 45%, respectively (Supplementary File S1 Figure S3). The bacteriolytic proteases belong to these groups. In this pool, we found a gene (UOF12968.1) whose expression increased 8-fold—almost the same as an increase in the expression of the Blp and L5 genes (Figure 2a), which contribute significantly to the bacteriolytic activity of the culture. This gene encodes a new, previously unexplored enzyme. We called the new enzyme Serp3.

We also analyzed an increase in the expression of genes of the known antifungal enzymes (Table 1, Figure 2) not previously annotated in *L. capsici*. As a result, the genes of the enzymes GluA, GluB, GluC, and chitinase were annotated. These enzymes were identical to those of *L. enzymogenes* N4-7 glucanases GluA, GluB, GluC [9] and *Lysobacter* sp. MK9-1 chitinase [10] by 92.0%, 86.2%, 87.7%, and 79.5%, respectively. The GluA and GluB glucanase genes on RM medium significantly increased their expression levels (by 371 and 205 times, respectively) (Table 1, Figure 2a). On SYM medium, the expression of the genes of these enzymes increased only 2.0 and 1.8 times for GluA and chitinase, respectively (Table 1, Figure 2b). Thus, the antifungal activity observed on SYM medium is probably not related to the production of antifungal enzymes. This activity can be due to the production of antibiotics.

*L. capsici* has been shown to be capable of producing antibiotics and antimicrobial peptides [3,11,12], e.g., the heat-stable antifungal factor, HSAF [3]. We analyzed the genes of the biosynthetic pathways of the known antibiotics in strain VKM B-2533^T^, which increased their expression on the chosen media (Table 2).

As shown in Table 2, the biosynthetic non-ribosomal peptide synthetase/polyketide synthase gene involved in the biosynthesis of the antibiotic HSAF increased its expression on RM medium by 3.8 times and on SYM medium by 1.4 times. Thus, the antifungal activity can be due to the production of HSAF antibiotic on both culture media. Analysis also revealed an increase in the expression of genes involved in the biosynthesis of lanthipeptides. In cultivation on RM medium, the expression of these genes was higher. We also annotated the genes of several non-ribosomal peptide synthetases (UOF17373.1, UOF17381.1, UOF17380.1), whose expression did not change in the cultivation of *L. capsici* VKM B-2533^T^ on RM medium, but it increased on SYM medium (by 1.4, 2.4 and 1.7 times, respectively). The functional significance of these synthetases is currently unknown.

Thus, the results confirm that the antimicrobial activity of *L. capsici* VKM B-2533^T^ is due to bacteriolytic enzymes, antibiotics and antimicrobial peptides. The main aim of the transcriptomic study was to identify the genes of the bacteriolytic enzymes. They include the genes of the known *Lysobacter* bacteriolytic enzymes (Table 1) as well as those of putative bacteriolytic enzymes that increased their expression levels. The earlier identified bacteriolytic enzymes comprise those not yet characterized: Serp, Serp6 and N-acetylglucosaminidase. The gene of a new enzyme Serp3 with putative bacteriolytic activity was annotated.

For isolation and further characterization, we chose the earlier unexplored proteases Serp and Serp3. Expressions of the genes of these enzymes increased on RM medium by 3.2 and 8.1 times, respectively (Table 1, Figure 2a).

### 2.2. Isolation and Characterization of L. capsici VKM B-2533^T^ Bacteriolytic Enzymes

**Figure 4:**
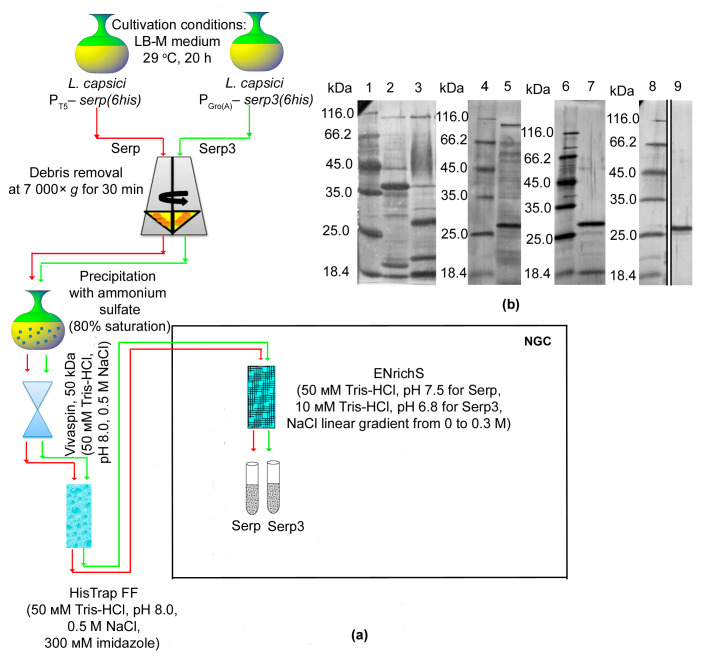
Purification of bacteriolytic enzymes Serp and Serp3 from the culture fluid of *L. capsici* expression strains. (**a**) Serp and Serp3 purification schemes. Red arrows indicate the Serp purification scheme; green arrows indicate the Serp3 purification scheme. (**b**) SDS-PAGE: 1, 4, 6 and 8, markers; 2, the culture fluid of wild-type *L. capsici* (Supplementary File S1 Figure S5); 3, the culture fluid of *L. capsici* P_Gro(A)_–*serp3*(*6his*) (Supplementary File S1 Figure S5); 5, the culture fluid of *L. capsici* P_T5_–*serp*(*6his*) (Supplementary File S1 Figure S6); 7, purified Serp3 of *L. capsici* P_Gro(A)_–*serp3*(*6his*) (0.18 μg) (Supplementary File S1 Figure S7); 9, purified Serp of *L. capsici* P_T5_–*serp* (0.35 μg) (Supplementary File S1 Figure S8). A 12 μL amount of the preparations was applied to the electrophoresis.

To isolate the new bacteriolytic enzymes Serp and Serp3, we produced, respectively, *L. capsici* P_T5_–*serp*(*6his*) and *L. capsici* P_Gro(A)_–*serp3*(*6his*) expression strains (Methods, Supplementary File S1 Figure S4). The strains were cultivated on LB-M medium with Gm 20 at 29 °C for 20 h. Next, the proteins of the culture fluid were analyzed electrophoretically; major bands were observed in the region of MWs 26 kDa and 30 kDa, corresponding to the calculated MWs of the bacteriolytic enzymes Serp and Serp3, respectively (Figure 4b).

A purification scheme was worked out for each enzyme (Methods, Figure 4a). As the result, bacteriolytically active proteins were isolated in an electrophoretically homogeneous form (Figure 4b) and were characterized. Extra bands in the preparations of bacteriolytic proteases Serp and Serp3 are associated with autolytic processes.

**Figure 5:**
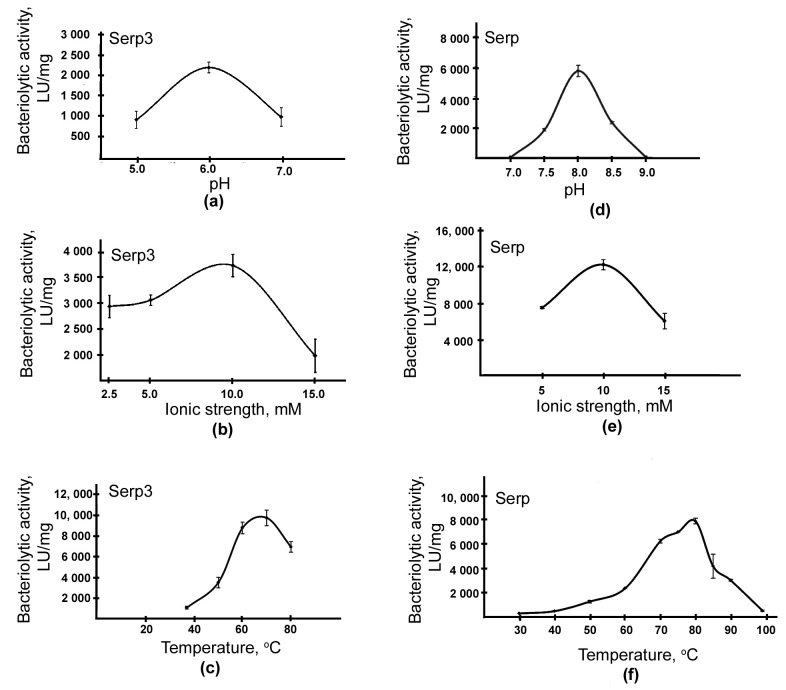
Optimal conditions of hydrolysis of autoclaved *S. aureus* 209P cells by bacteriolytic enzymes Serp3 (**a**–**c**) and Serp (**d**–**f**).

To study the optimal conditions for the bacteriolytic activities of Serp and Serp3 enzymes, autoclaved cells of *S. aureus* 209P were used as substrate (Figure 5).

Figure 5 shows that the maximum bacteriolytic activity of the enzymes is manifested at a Tris-HCl buffer concentration of 10 mM (Figure 5b,e); the optimal pH values for Serp3 are 6.0 (Figure 5a); for Serp, they are 8.0 (Figure 5d). Both enzymes are characterized by high reaction temperature optima of 80 °C and 70 °C for Serp (Figure 5f) and Serp3 (Figure 5c), respectively.

The specificity of action of the enzymes with respect to protein substrates and target cells was studied at 37 °C in order to assess the prospects of their use in medicine. Table 3 presents the results of studying the action of the bacteriolytic enzymes Serp and Serp3 with respect to autoclaved target cells.

As shown in Table 3, both enzymes were active with respect to *Micrococcus luteus* Ac-2230^T^, *Proteus vulgaris* H-19, *Pseudomonas aeruginosa* and *S. aureus* 209P. In relation to these cells, the activity of Serp3 was significantly higher than that of Serp. At the same time, Serp3 was inactive with respect to *Bacillus cereus* 217 cells, unlike Serp. Both enzymes were inactive against *Kocuria rosea* Ac-2200^T^ cells.

**Figure 6:**
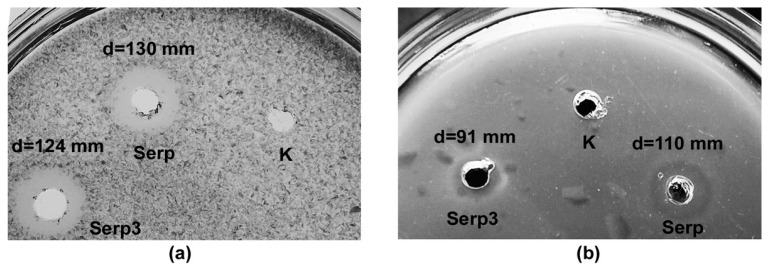
Specificity of action of the bacteriolytic enzymes Serp and Serp3 with respect to protein: (**a**) azofibrin; (**b**) casein. K, 10 mM Tris-HCl, pH 8.0. The diameter of the zones was measured from the center of the well to the edge of the proteolysis zone.

The specific proteolytic activities of Serp and Serp3 were 2.17 ± 0.23 and 5.98 ± 0.90 PE/mg, respectively. This confirms that the newly discovered enzymes are bacteriolytic proteases, which, in addition to bacterial cell walls, can hydrolyze protein substrates. The specificity of action of Serp and Serp3 with respect to casein, azofibrin, hemoglobin, gelatin, elastin, and collagen was studied by the spot test method (Table 4, Figure 6).

It was shown that Serp and Serp3 hydrolyzed only casein (Figure 6a) and azofibrin (Figure 6b).

Thus, we isolated and characterized new bacteriolytic proteases Serp and Serp3 of *L. capsici* VKM B-2533^T^. Subsequently, these proteins will be studied as a basis for creating antimicrobial drugs with proteolytic properties.

## 3. Discussion

The main aim of this study was to search for new bacteriolytic enzymes in *L. capsici*. While earlier, we have developed complex purification schemes for the isolation of bacteriolytic enzymes from *L. capsici* culture fluid, in this work, we searched for the genes of such proteins using a transcriptomic approach. The main idea was to search for the genes of bacteriolytic enzymes that increased their expression levels in the cultivation of *L. capsici* VKM B-2533^T^ on RM medium conducive to the production of such proteins. Medium 5/5 was chosen as a control in cultivation on which the lytic activity was practically absent. We assumed that the expression of genes of the major bacteriolytic enzymes would be decreased on this medium. This idea proved successful, and the genes of the known and putative bacteriolytic enzymes were annotated (Table 1, Supplementary File S2).

Among the genes that increased their expression levels on RM medium were genes of the known bacteriolytic enzymes, which were previously identified in strain VKM B-2533^T^ (Table 5). The genes of the L5 and L4 enzymes were annotated in strain VKM B-2533^T^ for the first time.

The greatest increase in expression was shown for the enzymes Blp, L1 and L5 (7, 39 and 6 times, respectively). Other bacteriolytic enzymes also increased their expression levels on RM medium. Bacteriolytic enzymes are known to be synergic [4], so even a slight increase in the gene expression of individual bacteriolytic enzymes can lead to a significant increase in the overall bacteriolytic activity as a whole.

Transcriptomic analysis revealed that among the entire pool of genes annotated as proteolytic enzymes, the greatest increase in expression was observed for metalloprotease and serine protease genes, 46% and 45%, respectively (Supplementary File S1 Figure S3). These are exactly the groups of enzymes to which bacteriolytic proteases belong. From this pool, we chose the UOF12968.1 gene annotated as a serine protease. This gene increased its expression by eight times, which is comparable with an increase in the expression of the Blp and L5 genes that contribute significantly to the overall bacteriolytic activity of the culture. We called the new enzyme Serp3.

For isolation and further characterization, we chose two new serine proteases not characterized previously, Serp and Serp3. The proteins were isolated from the culture fluid of the expression strains and characterized. The maximum bacteriolytic activity of the enzymes was shown to be manifested at low ionic strength of 10 mM Tris-HCl. Serp and Serp 3 showed maximum activities at high temperatures of 80 and 70 °C, respectively. pH 8.0 was optimal for Serp; slightly acidic conditions of pH 6.0 were optimal for Serp3. Both enzymes hydrolyzed dead cells of *M. luteus* Ac-2230^T^, *P. vulgaris* H-19, *P. aeruginosa* and *S. aureus* 209P, and Serp also digested cells of *B. cereus* 217. Both enzymes did not hydrolyze *K. rosea* Ac-2200^T^ cells. The enzymes had proteolytic activities on casein and azofibrin.

Thus, we characterized two new bacteriolytic enzymes in *L. capsici*. To date, six out of the nine known bacteriolytic enzymes of this species have been characterized (Table 5). *L. capsici* is the only bacterium for which such a large number of extracellular bacteriolytic enzymes have been described. Extracellular bacteriolytic enzymes are also known for *P. aeruginosa* Paks I (LasA) [25], *S. simulans* bv. *staphylolyticus* (lysostaphin) [26], *S. capitis* EPK1 (Ale-1) [27], *Streptococcus equi* subsp. *zooepidemicus* 4881 (zoocin) [28], *Enterococcus faecalis* DPC5280 (enterolysin A) [29], *S. milleri* NMSCC (millericin B) [30], and *Pseudoalteromonas* sp. CF6-2 (pseudoalterin) [31].

As *L. capsici* also has an antifungal activity, to search for genes of antifungal enzymes, we chose SYM medium, since cultivation on this medium leads to activity manifested maximally. The genes of the known antifungal agents, identical to the *L. enzymogenes* N4-7 genes of GluA, GluB, GluC glucanases [9] by 92.0, 86.2, 87.7%, respectively, were annotated in strain VKM B-2533^T^. The chitinase gene, 79.5% identical to the *Lysobacter* sp. MK9-1 chitinase gene, was also annotated [10]. What was unexpected was that these genes increased their expression levels on RM medium, whereas we expected this increase on SYM medium. This indicates that enzymes or antibiotics unknown to us can contribute to the antifungal activity of *L. capsici* on SYM medium.

The most investigated antifungal *Lysobacter* antibiotic is HSAF [32,33]. Analysis of the genes of the HSAF biosynthetic pathway revealed the biosynthetic non-ribosomal peptide synthetase/polyketide synthase gene, which increased its expression on RM medium by 3.8 times and on SYM medium by 1.4 times. Thus, the antifungal activity of the strain can be conditioned by both antifungal enzymes and HSAF. However, it is obvious that besides the antifungal agents known in *Lysobacter*, there are also unknown ones that have yet to be identified. Analysis also revealed an increase in the expression of genes involved in the biosynthesis of lanthipeptides. In cultivation on RM medium, the expression of biosynthetic genes of these agents is more pronounced. On the whole, lanthipeptides possess antibacterial and antifungal activities [34]; however, they have not been isolated from *Lysobacter* bacteria before. We also annotated the genes of several non-ribosomal peptide synthetases (UOF17373.1, UOF17381.1, UOF17380.1), which did not change their expression on RM medium but reliably increased it on SYM medium (by 1.4, 2.4 and 1.7 times, respectively). The functional significance of these synthetases is not currently known.

As our interest is focused on the secreted antimicrobial agents of *Lysobacter*, we also analyzed the genes of the known secretory pathways that increased their expression levels in response to an increase in the expression of secreted products (Supplementary File S1 Table S2). T2SS is involved in the secretion of proteases, lipases and chitinases [35,36]. In the cultivation of *L. capsici* VKM B-2533^T^ on RM medium, the expression of some of the genes of this secretory pathway was found to increase, while on SYM medium, it either did not change or decreased. This result is logical, because in the cultivation of the strain on RM medium, the expression of the genes of bacteriolytic and antifungal enzymes did increase significantly. We also paid attention to T4SS and T6SS involved in antimicrobial interaction, as it is known that these secretion pathways deliver antibacterial (T4SS and T6SS) and antifungal (T6SS) effectors directly to the target cell [37,38,39]. We found an increase in the expression of the T4SS and T6SS genes in the cultivation of *L. capsici* VKM B-2533T on RM and SYM media, respectively. In the cultivation of *L. capsici* VKM B-2533^T^ on RM medium, the gene of T4SS effector—a metalloprotease of the M23B family (UOF17279.1)—increased its expression by 1.5 times. This gene is 45.3% identical to the earlier discovered XAC2609 effector from *Xanthomonas citri* [40], for which an antibacterial effect has been established against living *B. subtilis* cells and *M. luteus* peptidoglycan. An increase in the expression of T4SS genes, in particular those responsible for the assembly and secretion of pili, was also found. This can play an important role both in cell motility and in the realization of its lytic potential, which has been shown earlier for *L. enzymogenes* OH11 [41] and *L. capsici* AZ78 [42]. The increase in the level of expression of T6SS genes in the cultivation of *L. capsici* VKM B-2533^T^ on SYM medium is noteworthy; herewith, on RM medium, the gene expression either decreased or else did not change. This can be indicative of the involvement of T6SS in the secretion of antifungal factors. Earlier, for *L. gummosus* 3.2.11, the presence of the T6SS secretory pathway has been shown, and an assumption has been made about the main effectors (lytic transglycosylase, OmpA family, peptidoglycan binding domain), which are associated with the T6SS gene cluster [43]. However, the physiological purpose of these effectors has not been determined. The antifungal effect of T6SS effectors has been first proven for Tfe1 and Tfe2 of *Serratia marcescens* Db10 [44]. 

Thus, the results of this transcriptomic study confirm the significant antimicrobial potential of *L. capsici*. Our further research will aim to establish new antimicrobial agents and to investigate the role of secretory pathways in the microbial antagonism of *Lysobacter* bacteria.

## 4. Materials and Methods

### 4.1. Bacterial Strains and Cultivation Conditions

A list of the bacterial strains and plasmids used is given in Table 6.

For the cultivation of *L. capsici* VKM B-2533^T^ cells, media of the following composition (g/l) were used: RM glucose, 5.0; peptone, 2.0; yeast extract, 2.0; Na_2_HPO_4_·12H_2_O, 4.2; KH_2_PO_4_, 1.0; KCl, 0.6; MgSO_4_·7H_2_O, 5.0; FeSO_4_·7H_2_O, 0.1; pH 7.0 [5]; SYM sucrose, 10.0; yeast extract, 5.0, pH 7.0 [48]; 5/5, developed at the IBPM RAS, yeast extract, 1.0; soybean extract, 30.0; tryptone, 5.0; aminopeptide, 60.0, pH 7.2. The *L. capsici* expression strains were cultivated on modified LB-M medium (g/L): peptone, 5.0; yeast extract, 5.0; NaCl, 5.0, pH 7.5. The strains were cultivated at 29 °C with stirring (205 rpm).

The *E. coli* strain XL1-Blue was grown on LB medium (g/L): tryptone, 10; yeast extract, 5; NaCl, 10; pH 7.0 at 37 °C. 

The test cultures of *S. aureus* 209P, *M. luteus* Ac-2230^T^, *K. rosea* Ac-2200^T^, *B. cereus* 217, *P. aeruginosa* and *P. vulgaris* H-19 were cultivated on medium 5/5 at 29 °C for 18 h. Mycelial fungi *Fusarium solani* and *Sclerotium sclerotiorum* were cultivated on agarized medium wort at 29 °C.

### 4.2. Isolation of RNA

Strain *L. capsici* VKM B-2533^T^ was cultivated in three biological replicates on RM, SYM and 5/5 media for 19 h. Then, 2.0 a.u. of the culture was sampled from each flask (for a total of 9 preparations). Further on, the cells were harvested by centrifugation at 7000× *g* for 15 min. A RiboPure RNA Purification Kit (Thermo Scientific, Waltham, MA, USA) was used to isolate the total bacterial RNA in accordance with the manufacturer’s recommendation. The quality of the RNA preparations was assessed electrophoretically in 4% PAG with 8 M urea as well as by capillary electrophoresis using a Bioanalyzer 2100 (Agilent, Santa Clara, CA, USA). Ribosomal RNA was removed using a Ribo-Zero Plus rRNA Depletion Kit (Illumina, San Diego, CA, USA). cDNA synthesis with the subsequent preparation of libraries was carried out using a NEBNext Ultra II Directional RNA Library Prep Kit for Illumina (New England Biolabs, Ipswich, MA, USA). The library was sequenced on the Illumina HiSeq 4000 system (Illumina, San Diego, CA, USA) to obtain 151 bp reads. 

### 4.3. RNA-Seq Data Analysis

The quality of reads was controlled using FastQC v0.12.1 [49]. Adapter sequences and low-quality regions in raw reads were removed using Trimomatic v0.39 [50]. The clean reads were mapped on the *L. capsici* VKM B-2533^T^ genome (GenBank access No. CP094357.1) using the Bowtie2 v2 program v2.5.1 [51]; the mapped reads were counted using the featureCounts v2.0.4 [52]. The DESeq2 v1.34.0 package was used to assess differential gene expression [53]. Medium 5/5 was used as a control. A gene was assumed to change its expression at an adjusted *p*-value (*p*_adj_ < 0.05). NRPS/PKS clusters in *Lysobacter* genome sequences were identified using AntiSmash [54]. 

### 4.4. Molecular Genetic Kits and Equipment

All molecular genetic procedures were performed in accordance with Sambrook and Russell’s manual [55]. Restriction endonucleases, alkaline phosphatase, T4 DNA ligase, and T4 polynucleotide kinase were used (Thermo Fisher Scientific, Waltham, MA, USA). The PCR analysis was performed using the Q5 DNA polymerase (New England Biolabs, Ipswich, MA, USA) on a MiniAmp (Thermo Fisher Scientific, Waltham, MA, USA) in accordance with the manufacturer’s recommendation. The PCR reactions (total volume, 50 µL) were conducted under the following conditions: 200 mM dNTPs, 0.5 µM forward and reverse primers, *L. capsici* VKM B-2533^T^ DNA, 0.02 U/µL Q5 high-fidelity DNA polymerase (New England Biolabs, Ipswich, MA, USA) in 1 × reaction buffer containing 2 mM MgCl_2_. The thermo cycles were programmed according to the manufacturer’s protocol: initial denaturation at 98 °C for 30 s followed by 25 cycles at 98 °C for 10 s; annealing temperature 60 °C for 20 s, 72 °C for the time determined by amplicon length (extension times are 30 s per kb), and a final extension at 72 °C for 2 min. The list of oligonucleotides is given in Table 7.

DNA electrophoresis was performed in 0.8% agarose gel in a TAE buffer containing 0.5 mg/mL ethidium bromide. DNA was visualized in gel at 354 nm using a Bio-Print ST4 system (Vilber lourmat, Collégien, France). A QIAquick Gel Extraction Kit (Qiagen, Germantown, MD, USA) was used to extract DNA from the gel. A diaGene Kit (Diaem, Moscow, Russia) was used to isolate plasmids from *E. coli* XL1-Blue. A QIAamp DNA Mini Kit (Qiagen, Germantown, MD, USA) was used to isolate genomic DNA from *L. capsici* B-2533^T^. The quality and quantity of DNA preparations were evaluated electrophoretically in 0.8% agarose gel and on a NanoDrop OneC instrument (Thermo Fisher Scientific, Waltham, MA, USA). The transformation of highly competent *E. coli* XL1-Blue cells by a ligation mixture was performed by the RbCl method [56]. The electroporation of *L. capsici* VKM B-2533^T^ cells with constructed plasmids (Table 6) was completed in accordance with the Lin method with a modification [22] on a MicroPulser Electroporator (Bio-Rad, Hercules, CA, USA).

### 4.5. Construction of Plasmids and Production of L. capsici Expression Strains

pBBR1-MCS5 P_GroEL(A)_–*serp3*(*6his*): the amplicon, which was obtained as a result of the PCR with specific primers Serp3_HindIII (for) and Serp3_BamHI (rev) to the *serp3* gene with the genomic DNA of *L. capsici* VKM B-2533^T^ (amplicon size, 1233 bp), was ligated into the earlier constructed plasmid pBBR1-MCS5 P_GroEL(A)_–*gfp* treated by the HindIII/BamHI restriction sites. The ligation mixture was transformed into *E. coli* XL1-Blue. The isolated plasmid pBBR1-MCS5 P_GroEL(A)_–*serp3*(*6his*) was electroporated into competent *L. capsici* cells. The selection of *L. capsici* P_Gro(A)_–*serp3*(*6his*) clones was performed on agarized LB-M1 medium with Gm. The absence of mutation in the assembled construct was validated by sequencing using primers Gro_KpnI (for) and Term_XbaI (rev).

pBBR1-MCS5 P_T5_–*serp*(*6his*): the amplicon obtained by the PCR with specific primers Serp_BamHI (for) and Serp_HindIII (rev) to the *serp* gene with *L. capsici* VKM B-2533^T^ genomic DNA (amplicon size, 1395 bp) was ligated into the earlier constructed plasmid pBBR1-MCS5 P_T5_–*gfp* treated by the BamHI/HindIII restriction sites. The ligation mixture was transformed into *E. coli* XL1-Blue. The isolated plasmid pBBR1-MCS5 P_T5_–*serp*(*6his*) was electroporated into *L. capsici* competent cells. The selection of *L. capsici* P_T5_–*serp*(*6his*) clones was carried out on agarized LB-M1 medium with Gm. The absence of mutation in the assembled structure was validated by sequencing using primers T5_KpnI (for) and T5_XbaI (rev) (Evrogen, Moscow, Russia).

### 4.6. Purification of Bacteriolytic Enzymes

Bacteriolytic enzymes Serp and Serp3 were isolated from the culture fluid of the expression strains. Strains *L. capsici* P_Gro(A)_–*serp3*(*6his*) and *L. capsici* P_T5_–*serp*(*6his*) were cultivated in 600 mL of LB-M with 20 μg/mL of Gm for 20 h. The cells were then precipitated by centrifugation at 7000× *g* for 30 min at 4 °C. From the resulting culture fluid, proteins were precipitated with ammonium sulfate of 80% saturation, which was followed by centrifugation at 25.960× *g*. Furthermore, an individual purification scheme was developed for each bacteriolytic enzyme using the NGC chromatographic system (Bio-Rad, Hercules, CA, USA). At the first stage, the protein precipitate after (NH_4_)_2_SO_4_ was dissolved in 50 mM Tris-HCl, pH 8.0, and dialyzed against the same buffer with 0.5 M NaCl. Then, the mixture was centrifuged using Vivaspin 20, 50 kDa (Sartorius, Goettingen, Germany) to remove the exopolysaccharide. The resulting filtrate was applied onto a His trap FF column (GE Healthcare, Chicago, IL, USA) equilibrated with 50 mM Tris-HCl, pH 8.0, with 0.5 M NaCl and 3 mM imidazole. The lytically active fractions were combined and dialyzed against 50 mM Tris-HCl, pH 7.5, or 10 mM Tris-HCl, pH 6.8, in the case of Serp and Serp3, respectively. Furthermore, the protein preparations were applied to the ENrichS column equilibrated with the corresponding buffers. The proteins were eluted in a linear NaCl gradient from 0 to 0.3 M. The obtained Serp and Serp3 enzymes with concentrations of 0.030 mg/mL and 0.015 mg/mL, respectively, were stored in buffer at −20 °C. The homogeneity of the preparations of bacteriolytic proteases Serp and Serp3 was proved by gel filtration on a Hiload 16/60 column (Superdex 75).

The molecular weights were determined by the electrophoretic mobilities of the Serp and Serp3 proteins using GelAnalyzer software version 19.1 (http://www.gelanalyzer.com/?i=1) (accessed on 2 May 2023).

### 4.7. Electrophoresis of Proteins in PAG

The electrophoresis was performed in 12.5% PAG in the presence of sodium dodecyl sulfate according to the Laemmli method [57]. The samples were heated in a sample buffer (0.025 M Tris-HCl, 2% SDS, 10% glycerol, 0.7 M mercaptoethanol, bromophenol blue, pH 6.8) at 99 °C for 10 min. As markers, a mixture of protein standards (Thermo Fisher Scientific, Waltham, MA, USA) was used: β-galactosidase, 116.0 kDa; BSA, 66.2 kDa; ovalbumin, 45.0 kDa; lactate dehydrogenase, 35.0 kDa; REase Bsp981, 25.0 kDa; β-lactoglobulin, 18.4 kDa; lysozyme, 14.4 kDa. The electrophoresis in the concentrating gel was performed at 90 V; in the separating gel, it was performed at 180 V. Protein bands in the gel were revealed by staining with imidazole and ZnCl_2_ solutions [58].

### 4.8. Determination of Protein Concentration

The total protein concentration in the samples was measured by the Bradford method [59]. The reaction was carried out according to the protocol for the proprietary Coomassie reagent (Thermo Fisher Scientific, Waltham, MA, USA). The protein concentration was determined by a calibration curve constructed for an aqueous solution of BSA (Sigma, Ronkonkoma, NY, USA) within the range of 1 to 25 µg/mL.

### 4.9. Determination of Total Bacteriolytic Activity 

The total bacteriolytic activity was determined turbidimetrically, using autoclaved freeze-dried and living *S. aureus* 209P cells as substrate. A suspension of cells in 10 mM Tris-HCl, pH 8.0, with OD_540_ = 0.5 was prepared. Respective culture fluid preparations (5–50 μL) were added to the cell suspension. The final volume of the reaction mixture was 1 mL. The reaction mixture was incubated for 5–15 min at 37 °C.

The reaction was stopped by placing test tubes in ice. A decrease in the absorption of the suspension was recorded in the samples at 540 nm on a NanoDrop OneC instrument (Thermo Scientific, USA). The LU/mL value was calculated by the following formula:

[0.5 (initial OD_540_ of the suspension) − final OD_540_] × 1000 µL (total reaction volume)/[min (time of reaction) × µL (volume of sample) × 0.01 (correction coefficient for the OD reduction per min)].

Data from four independent biological experiments were processed for statistical analysis. All measurements were carried out in three biochemical replicates.

### 4.10. Determination of Optimal Conditions for the Bacteriolytic Activities of Serp and Serp3 to Be Exhibited

Autoclaved *S. aureus* 209P cells prepared in an appropriate buffer were used as substrate. The bacteriolytic activity was measured by the turbidimetric method as indicated above. The storage buffer of the enzyme preparation was used as a control.

To determine the optimal pH value for the hydrolysis of autoclaved *S. aureus* 209P cells by the Serp3 enzyme, use was made of 5-mM Britton–Robinson buffer, pH 5.0, 6.0, or 7.0. The reaction mixture contained 25–40 µL (0.38–0.60 µg) of the enzyme preparation. The mixture was incubated at 60 °C for 10–15 min. For Serp, we used 10 mM Tris-HCl, pH 7.0, 7.5, 8.0, 8.5, or 9.0. The reaction mixture contained 25 µL (0.19–0.75 µg) of the enzyme preparation. The mixture was incubated at 80 °C for 5 min.

To determine the effect of ionic strength of the solution for the manifestation of bacterioltic activity, for Serp3, we used a buffer of 2.5–15 mM Tris-HCl, pH 7.0. The reaction mixture contained 20 µL (0.30 µg) of the enzyme preparation. The mixture was incubated at 60 °C for 10–15 min. For Serp, 5–15 mM Tris-HCl buffer, pH 8.0, was used. The reaction mixture contained 5 µL (0.20 µg) of the enzyme preparation. The mixture was incubated at 80 °C for 5 min.

To determine the optimal value of the reaction temperature for Serp3, use was made of 10 mM Tris-HCl, pH 7.0. The reaction mixture contained 10–25 µL (0.06–0.16 µg) of the enzyme preparation. The mixture was incubated at a temperature of 37–80 °C for 10–45 min. For Serp, 10 mM Tris-HCl, pH 8.0, was used. The reaction mixture contained 25 µL (0.19–0.75 µg) of the enzyme preparation. The mixture was incubated at a temperature of 30–90 °C for 5–30 min. The specific activity of the enzymes was calculated as a ratio of LU per mg of protein. 

All measurements were carried out in three biochemical replicates.

### 4.11. Measurement of Proteolytic Activity

The proteolytic activities of the Serp3 and Serp enzymes were measured according to the Hall method [60]. For this, 0.2 mL of a solution of the Serp3 enzyme preparation (0.3 µg) and the Serp enzyme preparation (1.7 µg) was added to 0.2 mL of a 1% casein solution in 10 mM Tris-HCl, pH 7.0 and 10 mM Tris-HCl, pH 8.0, respectively. A casein solution without enzyme was poured into the control test tubes. The control and experimental test tubes were incubated for 30 min at 37 °C. The reaction was stopped by adding 0.8 mL of 5% TCA, 0.2 mL of the enzyme solution was added to the control test tube, and all samples were incubated again for 10 min at 37 °C to form a residue. The resulting residue was separated by centrifugation at 12,000× *g* for 5 min; in the supernatant, the absorption was determined at 280 nm. A standard curve was generated using tyrosine solutions. One unit of protease activity was defined as the amount of enzyme required to liberate 1 µmol of tyrosine per min under the experimental conditions. 

The PU/mL value was calculated by the following formula: [µmol tyrosine equivalents released) × 1200 µL (total reaction volume × 10 (dilution of sample)]/[30 min (time of reaction) × 200 µL (volume of sample)]. The specific activity of the enzymes was calculated as a ratio of LU per mg of protein. PU/mg was calculated as a ratio of LU per mg of protein. 

All measurements were carried out in three biochemical replicates.

### 4.12. Determination of the Specificity of Action of Bacteriolytic Enzymes against Protein Substrates and Autoclaved Bacterial Target Cells

The specificity of action of the bacteriolytic enzymes Serp3 and Serp was determined using casein, hemoglobin, azofibrin, gelatin, collagen and elastin as protein substrates by the spot test method. The substrates were dissolved in buffers optimal for each enzyme at a concentration of 10 g/l and poured into petri dishes with 1.5% agarose. In the cups, wells were made into which preparations of the bacteriolytic enzymes Serp 3 and Serp were introduced in a volume of 10 µL (0.12 µg and 0.80 µg, respectively). The dishes were incubated at 37 °C up to the appearance of clarification zones (up to 24 h). The diameters of the hydrolysis zones of the protein substrates were determined using AutoCAD 2012 software. All measurements were carried out in two biochemical replicates.

As target cells, use was made of autoclaved cells of *S. aureus* 209P, *M. luteus* Ac-2230^T^, *K. rosea* Ac-2200^T^, *B. cereus* 217, *P. aeruginosa* and *P. vulgaris* H-19 prepared as follows: after cultivation on medium 5/5 at 29 °C for 18 h, the cells were autoclaved at 1.0 atm for 60 min, precipitated at 12,000× *g* and washed twice with the buffer of an enzyme sample. Next, a suspension of cells in 10 mM Tris-HCl, pH 8.0, with OD_540_ = 0.5 was prepared. Then, 15–50 μL (0.05–0.16 µg) of bacteriolotyc protease Serp3 and 5–100 µL (0.20–8.00 µg) of Serp was added to the cell suspension. The final volume of the reaction mixture was 1 mL. The reaction mixture was incubated for 5–30 min at 37 °C. All measurements were carried out in three biochemical replicates. Then, a cell suspension was prepared to determine the total bacteriolytic activity by the formula given in Section 4.9.

### 4.13. Determination of Antifungal Activity by the Spot Test

Living cells of *F. solani* and *S. sclerotiorum* were used as substrates. A suspension of fungal cells was prepared from the seed material. An amount of 100 µL of the suspension was dispersed with a spatula onto petri dishes with agarized medium wort. Then, wells were made in the agar, and 30 µL of culture fluid preparations was poured into the wells. The dishes were incubated for 24–48 h at room temperature. The lytic activity was determined by the presence of a lysis zone at the site of sample application. The diameters of the hydrolysis zones of the protein substrates were determined using AutoCAD 2012 software. For statistical analysis, data of three independent biological experiments were obtained, each of which had two biochemical replicates.

## Figures and Tables

**Table 1:** Change of expression of genes encoding lytic enzymes.

Enzymes (Protein_Id)/(Locus_Tag)		Change of Expression *	
		RM	SYM
Bacteriolytic enzymes			
Protease Blp (UOF15870.1)/(IEQ11_04180)	7.0		1.5
Protease L1 (UOF13170.1)/(IEQ11_15580)	39.1		2.4
Protease L4 (UOF16892.1)/(IEQ11_09745)	2.9		0.6
Protease L5 (UOF13168.1)/(IEQ11_15570)	6.0		0.6
Protease Serp (UOF16681.1)/(IEQ11_08595)	3.2		0.7
Protease Serp6 (UOF14691.1)/(IEQ11_23755)	2.2		2.8
Protease Serp7 (UOF17397.1)/(IEQ11_12530)	4.2		1.7
N-acetylglucosaminidase (UOF15745.1)/(IEQ11_03495)	1.7		1.8
Antifungal enzymes			
Glucanase GluA (UOF14439.1)/(IEQ11_22400)	370.9		2.0
Glucanase GluB (UOF13513.1)/(IEQ11_17420)	204.6		0.6
Glucanase GluC (UOF16369.1)/(IEQ11_06885)	3.0		0.6
Chitinase (UOF12917.1)/(IEQ11_14225)	14.5		1.8

* Values ≥ 1, expression increase; ≤1, expression decrease.

**Table 2:** Differential expression of genes that can be involved in the biosynthesis of antifungal agents (at *p*_adj_ < 0.05).

Enzymes (Protein_Id)/(Locus_Tag)	Change of Expression of Genes	
	RM	SYM
**Secondary metabolites (antibiotics and peptides)**		
Class III lanthipeptide (UOF16985.1)/(IEQ11_10290)	7.7	4.0
Class III lanthionine synthetase LanKC (UOF16986.1)/(IEQ11_10295)	8.5	3.6
Class 2 lanthipeptide synthetase LanM family protein (UOF15613.1)/(IEQ11_02800)	1.7	3.5
Class 2 lanthipeptide synthetase LanM family protein (UOF15622.1)/(IEQ11_02845)	5.2	2.0
Non-ribosomal peptide synthetase (UOF17373.1)/(IEQ11_12390)	–	1.4
Non-ribosomal peptide synthetase (UOF17381.1)/(IEQ11_12435)	–	2.4
Non-ribosomal peptide synthetase (UOF17380.1)/(IEQ11_12430)	–	1.7
Class 2 lanthipeptide synthetase LanM family protein (UOF14422.1)/(IEQ11_22315)	3.1	2.2
Non-ribosomal peptide synthetase (UOF14553.1)/(IEQ11_23005)	1.5	–
Non-ribosomal peptide synthetase (UOF14554.1)/(IEQ11_23010)	1.8	–
HSAF biosynthetic non-ribosomal peptide synthetase/polyketide synthase (UOF16764.1)/(IEQ11_09055)	3.8	1.4

–, Statistically insignificant change of gene expression.

**Table 3:** Bacteriolytic activities of Serp and Serp3 with respect to autoclaved target cells.

	Bacterial Substrates	*Micrococcus luteus* Ac-2230^T^	*Kocuria rosea* Ac-2200^T^	*Bacillus cereus* 217	*Proteus vulgaris* H-19	*Pseudomonas aeruginosa*	*S. aureus* 209P
Enzymes		Bacteriolytic Activity, LU/mg					
Serp		7700 ± 346	0	1492 ± 142	2533 ± 126	806 ± 63	1422 ± 258
Serp3		24,700 ± 816	0	0	7114 ± 251	5536 ± 286	3562 ± 112

**Table 4:** Specificity of action of the bacteriolytic enzymes Serp and Serp3 with respect to protein substrates.

Bacteriolytic Enzymes	Casein	Azofibrin	Hemoglobin	Gelatin	Elastin	Collagen
Serp	+	+	–	–	–	–
Serp3	+	+	–	–	–	–

**Table 5:** Genes of the bacteriolytic enzymes, whose expression increased (at *p*_adj_ < 0.05).

Bacteriolytic Enzyme (Protein_Id)/(Locus_Tag)	Identity with Earlier Isolated *Lysobacter* Proteins	References
Blp (UOF15870.1)/(IEQ11_04180)	Isolated from *L. capsici* VKM B-2533^T^ and characterized	[6,13]
L1 (UOF13170.1)/(IEQ11_15580)	99.25% identical with L1 (ACZ72924.1) of *L. capsici* XL1 [14]; has been investigated	[15,16,17]
L5 (UOF13168.1)/(IEQ11_15570)	99.00% identical with L5 (ACZ72925.1) of *L. capsici* XL1; has been investigated but was identified in strain VKM B-2533^T^ for the first time	[18,19,20,21,22]
L4 (UOF16892.1)/(IEQ11_09745)	99.7% identical with L4 of *L. capsici* XL1; has been investigated	[23]
Serp (UOF16681.1) */(IEQ11_08595)	New enzyme, isolated from *L. capsici* VKM B-2533^T^, investigated in the present work	[6]
Serp6 (UOF14691.1)/(IEQ11_23755)	New enzyme, isolated from *L. capsici* VKM B-2533^T^, but has not been investigated	[6]
Serp7 (UOF17397.1)/(IEQ11_12530)	76.70% identical with lysin-specific serine protease (P15636.1) of *L. enzymogenes*, isolated from *L. capsici* VKM B-2533^T^, but has not been investigated as a bacteriolytic enzyme	[6,24]
Serp3 (UOF12968.1) */(IEQ11_14490)	New enzyme, identified and investigated in the present work	
N-acetylglucosaminidase (UOF15745.1)/(IEQ11_03495)	New enzyme, isolated from *L. capsici* VKM B-2533^T^, but has not been investigated	[6]

* bacteriolytic enzymes chosen for study.

**Table 6:** Strains and plasmids used.

Strains and Plasmids	Characteristic	Refs
*Lysobacter capsici* VKM B-2533^T^	Wild type	[45]
*L. capsici* P_T5_–*serp*(*6his*)	Strain *L. capsici* VKM B-2533^T^ with plasmid pBBR1-MCS5 P_T5_–*serp*(*6his*)	This work
*L. capsici* P_GroEL(A)_–*serp3*(*6his*)	Strain *L. capsici* VKM B-2533^T^ with plasmid pBBR1-MCS5 P_GroEL(A)_–*serp3*(*6his*)	This work
*Escherichia coli* XL1–Blue	*recA1 endA1 gyrA96 thi hsdR17 supE44 relA1 lac*/[F′::Tn10 *proAB+ lacI^q^ lacZ*Δ*M15 traD36*]	[46]
pBBR1-MCS5 *	Broad-host vector, Gm^R^	[47]
pBBR1-MCS5 P_T5_–*gfp*	Plasmid pBBR1-MCS5 with gene *gfp* under the regulation of bacteriophage T5 promoter	[8]
pBBR1-MCS5 P_GroEL(A)–_*gfp*	Plasmid pBBR1-MCS5 with gene *gfp* under the regulation of GroEL promoter of *L. enzymogenes* with modification	[8]
pBBR1-MCS5 P_T5_–*serp*(*6his*)	Plasmid pBBR1-MCS5 with gene *serp* and a tag of 18 nucleotides encoding His6-tag, under the regulation of bacteriophage T5 promoter	This work
pBBR1-MCS5 P_GroEL(A)_–*serp3*(*6his*)	Plasmid pBBR1-MCS5 with gene *serp3* and a tag of 18 nucleotides encoding His6-tag, under the regulation of GroE promoter of *L. enzymogenes* with modification	This work

* The plasmid was a kind gift from Dr. Yangyang Zhao (Institute of Plant Protection, Jiangsu Academy of Agricultural Sciences, Jiangsu Key Laboratory for Food Quality and Safety—State Key Laboratory Cultivation Base of the Ministry of Science and Technology, Nanjing, China).

**Table 7:** Oligonucleotides used in this work.

Oligonucleotides	Sequence	Goal
**Oligonucleotides used for cloning**		
Serp_BamHI (for)	GGATCCATGATCCGCAAGAACGCACTTTG	To amplify gene *serp*(*6his*) 1395 bp long with DNA of *L. capsici* VKM B-2533^T^
Serp_HindIII (rev)	AAGCTTTCAATGATGATGATGATGATGGGGATTGAAATAGCTCGACACG	
Serp3_HindIII (for)	AAGCTTATGCGTAAGTTCAGTTTGTCGATCCTC	To amplify gene *serp3*(*6his*) 1233 bp long with DNA of *L. capsici* VKM B-2533^T^
Serp3_BamHI (rev)	GGATCCTTAATGATGATGATGATGATGCGGACTGGTGCG	
**Oligonucleotides used for sequencing**		
T5_KpnI (for)	GGTACCGTGCCACCTGACGTCTAAG	To confirm absence of mutation in the cloned sequence in the assembled pBBR1-MCS5 P_T5_–*serp*(*6his*) constructs
T5_XbaI (rev)	TCTAGACTGAAAATCTCGCCAAGCTAGC	
Gro_KpnI (for)	GGTACCCGGACCGACGCCTGTCA	To confirm absence of mutation in the cloned sequence in the assembled pBBR1-MCS5 pBBR1-MCS5 P_GroEL(A)_–*serp3*(*6his*) constructs
Term_XbaI (rev)	TCTAGAAGAGTTTGTAGAAACGCAAAAAGGC	

## Data Availability

All data and materials related to the study are available upon request.

## Supplementary Materials

The supporting information can be downloaded at: https://www.mdpi.com/article/10.3390/ijms241411652/s1.
