# Supplementary material for: Transcriptomic Analysis Followed by the Isolation of Extracellular Bacteriolytic Proteases from Lysobacter capsici VKM B-2533T
Source: Int J Mol Sci. 2023 Jul 19;24(14):11652. doi: 10.3390/ijms241411652 (PMC10380237; doi:10.3390/ijms241411652)
Supplement: Supplementary file 1 [file ijms-24-11652-s001.zip › Supplementary File S1.pdf]

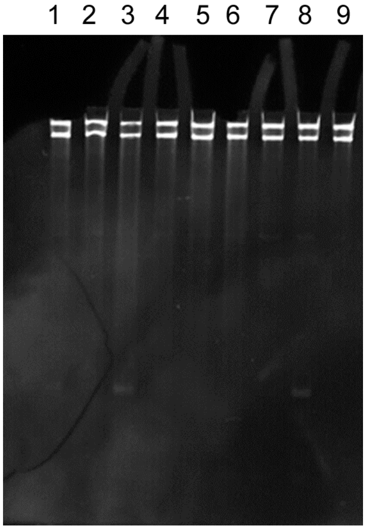

Supplementary File S1 Figure S1. Electrophoregram of the preparations of *L. capsici* VKM B-2533^{T} total RNA in PAG with 8 M urea : 1, 2, 3 – 5/5 medium (3 biological replicates); 4, 5, 6 – SYM medium (3 biological replicates); 7, 8, 9 – RM medium (3 biological replicates).

Supplementary File S1 Table S1. RNA-Seq statistics.

Sample	No. of reads before trimming	No. of reads after trimming	No. of mapped reads	No. of assigned reads
5-5 rep. 1	10,341,506	10,309,383	10,185,804	5,838,564
5-5 rep. 2	11,582,325	11,534,475	11,321,540	6,606,217
5-5 rep. 3	11,827,240	11,783,722	11,560,481	7,187,329
RM rep. 1	14,178,017	14,133,255	13,969,665	11,463,880
RM rep. 2	13,461,388	13,381,702	13,067,723	10,358,674
RM rep. 3	11,705,472	11,661,745	11,344,261	8,715,177
SYM rep. 1	10,778,190	10,732,726	10,600,405	6,438,004
SYM rep. 2	11,602,110	11,551,923	11,421,728	6,514,277
SYM rep. 3	9,965,989	9,908,466	9,689,545	7,929,300

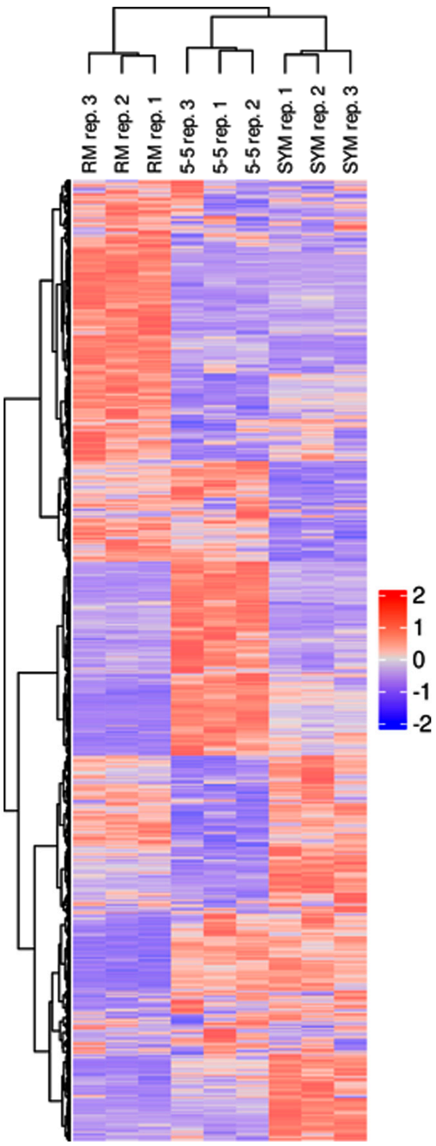

Supplementary File S1 Figure S2. Heatmap of transcript counts for 5-5, RM, and SYM media.

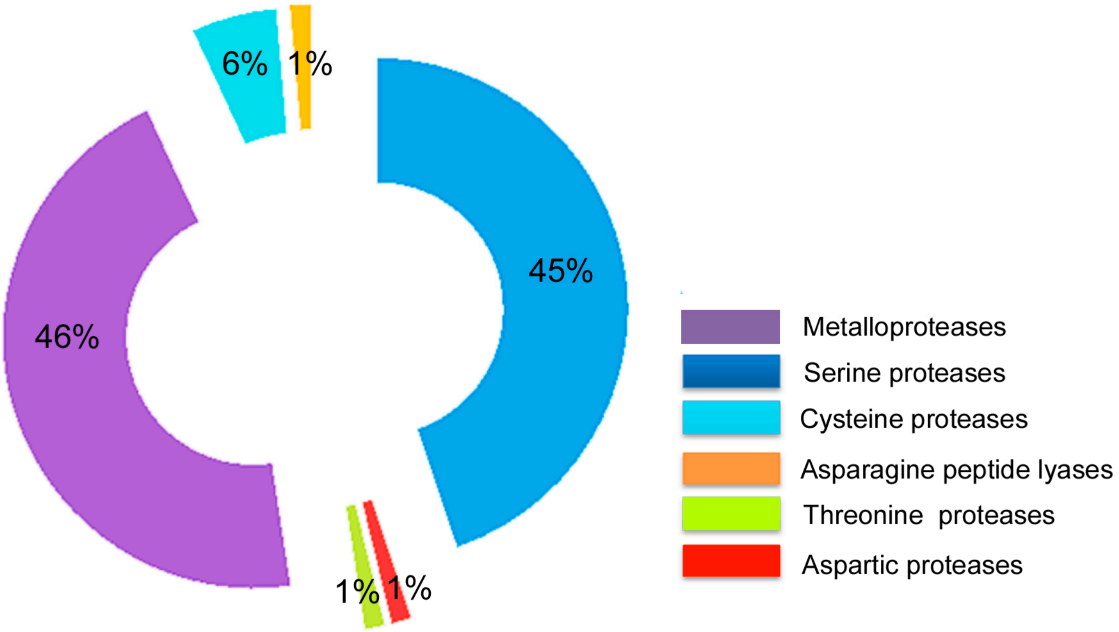

Supplementary File S1 Figure S3. Proteases whose genes increased their expression. The proteases were annotated using the Merops database (https://www.ebi.ac.uk/merops/).

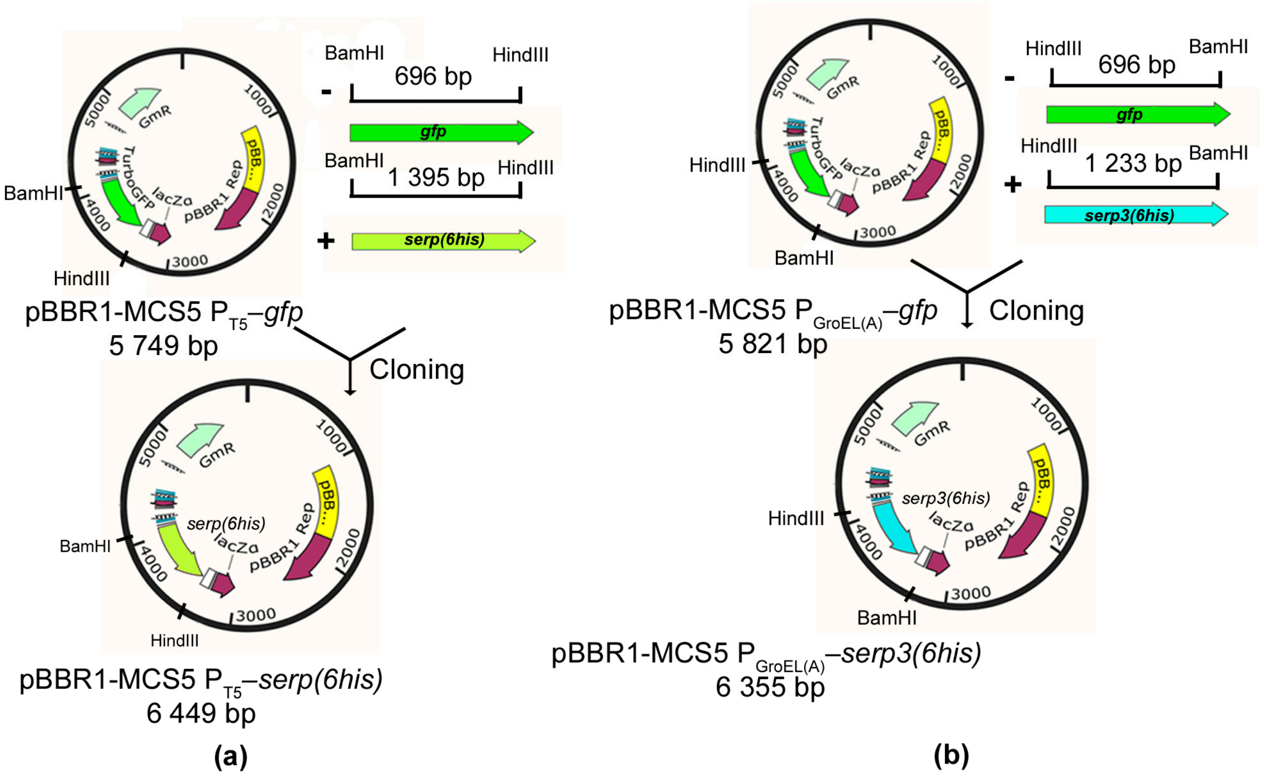

Supplementary File S1 Figure S4. Scheme of constructing expression vectors pBBR1-MCS5 P_{T5}–*serp(6his)* **(a)**; pBBR1-MCS5 $P_{GroEL(A)}$–*serp3(6his)* **(b).**

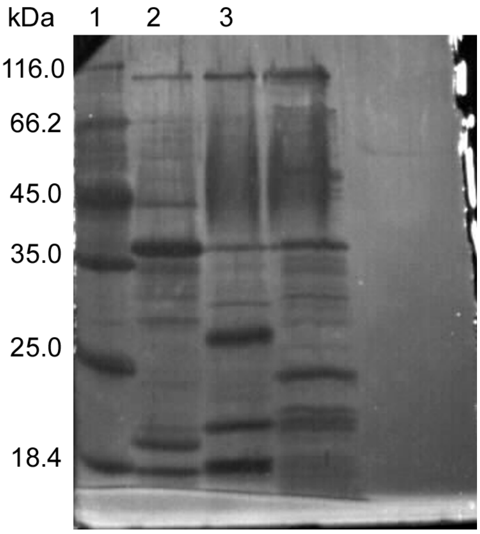

Supplementary File S1 Figure S5. Original gel images for Figure 4: lane 1 corresponds to M of Figure 4; lanes 2 and 3, to samples of culture fluid of wild-type *L. capsici* and *L. capsici* $P_{Gro(A)}$–*serp3(6his),* respectively, of Figure 4.

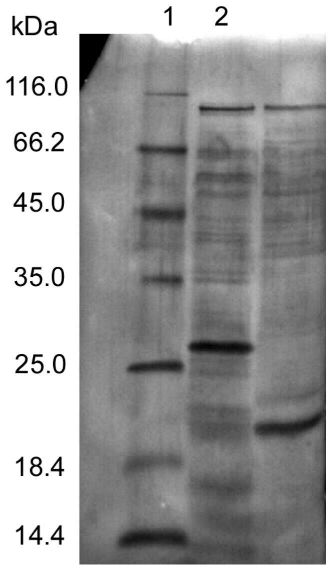

Supplementary File S1 Figure S6. Original gel images for Figure 4: lane 1 corresponds to M of Figure 4; lane 2 corresponds to the sample of culture fluid of *L. capsici* P_{T5}–*serp(6his)* of Figure 4.

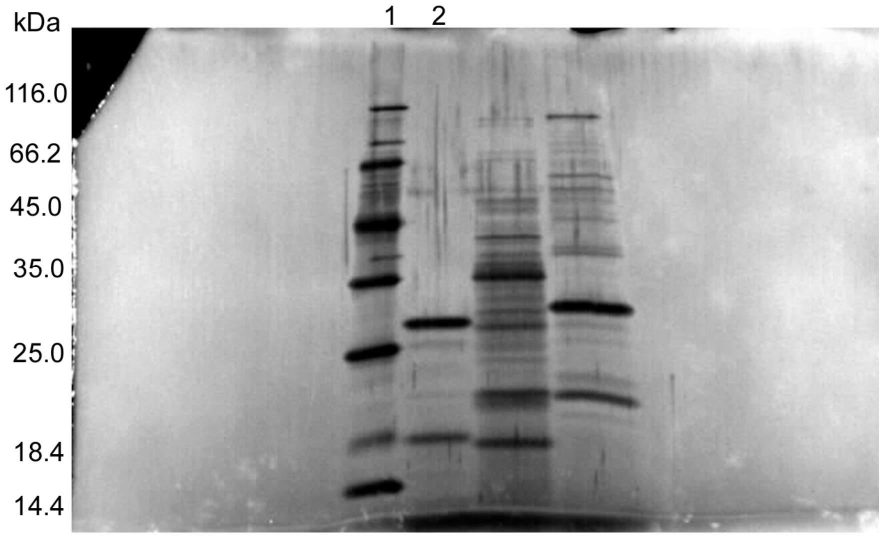

Supplementary File S1 Figure S7. Original gel images for Figure 4: lane 1 corresponds to M of Figure 4; lane 2 corresponds to the sample of purified Serp3 of *L. capsici* $P_{Gro(A)}$–*serp3(6his)* of Figure 4.

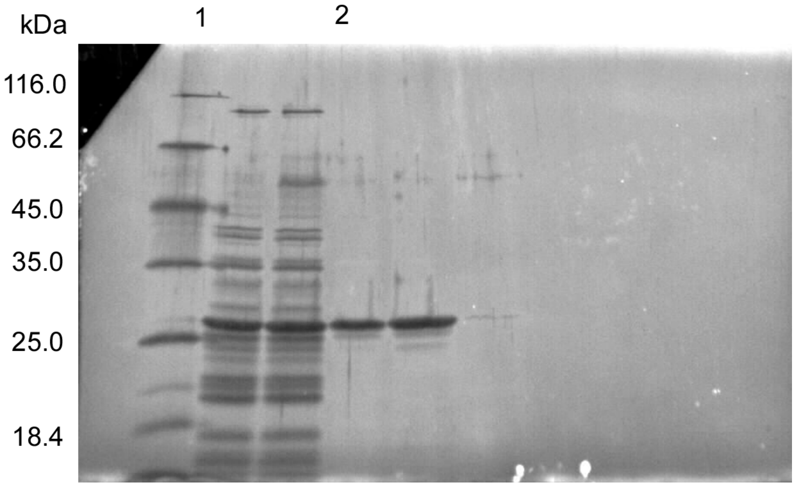

Supplementary File S1 Figure S8. Original gel images for Figure 4: lane 1 corresponds to M of Figure 4; lane 2 corresponds to the sample of purified Serp of *L. capsici* P_{T5}–*serp(6his)* of Figure 4.

Supplementary File S1 Table S2. Differential expression of genes, which can be involved in secretion of antimicrobial agents (at $p_{adj} < 0.05$).

Secretory pathways		
	**RM**	**SYM**
**T2SS**		
type II secretion system protein GspG (UOF16568.1)/(IEQ11_07940)	0.5	–
type II secretion system F family protein (UOF13912.1)/(IEQ11_19580)	1.3	0.8
type II secretion system secretin GspD (UOF13961.1)/(IEQ11_19855)	1.4	–
general secretion pathway protein GspN (UOF17641.1)/(IEQ11_19860)	1.6	–

general secretion pathway protein GspK (UOF13963.1)/(IEQ11_19875)	0.8	0.7
type II secretion system ATPase GspE (UOF17576.1)/(IEQ11_19905)	1.5	–
GspH/FimT family pseudopilin (UOF16386.1)/(IEQ11_06975)	1.8	–
GspH/FimT family pseudopilin (UOF16480.1)/(IEQ11_07470)	1.9	0.5
**T4SS**		
Pilin (UOF16485.1)/(IEQ11_07495)	2.7	–
VirB3 family type IV secretion system protein (UOF17112.1)/(IEQ11_10985)	2.1	1.4
VirB4 family type IV secretion/conjugal transfer ATPase (UOF17113.1)/(IEQ11_10990)	1.4	–
type IV secretion system protein VirB6 (UOF17281.1)/(IEQ11_11905)	3.2	–
P-type DNA transfer ATPase VirB11 (UOF17110.1)/(IEQ11_10970)	1.4	–
TrbC/VirB2 family protein (UOF17111.1)/(IEQ11_10980)	1.8	1.8
type IV-A pilus assembly ATPase PilB (UOF13911.1)/(IEQ11_19575)	1.4	0.6
type IV pilus secretin PilQ (UOF15948.1)/(IEQ11_04620)	2.7	–
type IV pilus twitching motility protein PilT (UOF16146.1)/(IEQ11_05700)	1.8	0.7
type IV secretion protein Rhs (UOF16147.1)/(IEQ11_05710)	1.7	–
type IV pilus modification protein PilV (UOF16481.1)/(IEQ11_07475)	2.3	0.6
type IV secretion system protein VirB6 (UOF17116.1)/(IEQ11_11005)	–	1.5
type IVB secretion system protein IcmH/DotU (UOF17348.1)/(IEQ11_12265)	–	2.6
**T6SS**		
type VI secretion system membrane subunit TssM (UOF17332.1)/(IEQ11_12180)	0.5	2.9
type VI secretion system-associated protein TagF (UOF17333.1)/(IEQ11_12185)	0.5	2.5
type VI secretion system tip protein VgrG (UOF17335.1)/(IEQ11_12195)	–	4.5
type VI secretion system tip protein VgrG (UOF17347.1)/(IEQ11_12260)	–	4.3
type VI secretion system baseplate subunit TssK (UOF17349.1)/(IEQ11_12270)	1.3	2.4
hypothetical protein (UOF17351.1)/(IEQ11_12280)	0.7	1.6
type VI secretion system contractile sheath small subunit (UOF17352.1)/(IEQ11_12285)	–	2.1
type VI secretion system contractile sheath large subunit (UOF17353.1)/(IEQ11_12290)	0.6	2.1
type VI secretion system tube protein Hcp	0.2	1.7

(UOF17354.1)/(IEQ11_12295)		
type VI secretion system baseplate subunit TssE (UOF17355.1)/(IEQ11_12300)	–	2.4
type VI secretion system baseplate subunit TssF (UOF17356.1)/(IEQ11_12305)	0.6	2.7
type VI secretion system baseplate subunit TssG (UOF17357.1)/(IEQ11_12310)	0.6	2.3
type VI secretion system ATPase TssH (UOF17358.1)/(IEQ11_12315)	0.6	2.4
type VI secretion system tip protein VgrG (UOF17359.1)/(IEQ11_12320)	0.6	2.5

– Gene expression change is statistically insignificant
